# Genetic diversity and antimicrobial resistance of invasive, noninvasive and colonizing group B *Streptococcus* isolates in southern Brazil

**DOI:** 10.1099/acmi.0.000370

**Published:** 2022-06-01

**Authors:** Otto Henrique May Feuerschuette, Eduardo Venâncio Alves, Mara Cristina Scheffer, Ana Paula Pessoa Vilela, Fernando Hartmann Barazzetti, Henrique Miranda Feuerschuette, Ana Carolina Lobor Cancelier, Maria Luiza Bazzo

**Affiliations:** ^1^​ Hospital Universitário Professor Polydoro Ernani de São Thiago, HU/UFSC, Florianópolis, Brazil; ^2^​ Universidade do Sul de Santa Catarina – UNISUL, Tubarão, Brazil; ^3^​ Laboratório de Biologia Molecular, Microbiologia e Sorologias, HU/UFSC, Florianópolis, Brazil

**Keywords:** Group B *Streptococcus*;* Streptococcus agalactiae*, antimicrobial resistance, MLVA, genetic diversity

## Abstract

**Introduction.:**

Group B *Streptococcus* (GBS) is a human commensal bacterium that is also associated with infection in pregnant and non-pregnant adults, neonates and elderly people.

**Gap Statement.:**

The authors hypothesize that knowledge of regional GBS genetic patterns may allow the use of prevention and treatment measures to reduce the burden of streptococcal disease.

**Aim.:**

The aim was to report the genotypic diversity and antimicrobial sensitivity profiles of invasive, noninvasive urinary and colonizing GBS strains, and evaluate the relationships between these findings.

**Methodology.:**

The study included consecutive and non-duplicated GBS isolates recovered in southern Brazil from 2015 to 2017. We performed multiple-locus variable-number tandem repeat analysis (MLVA) and PCR analyses to determine capsular serotypes and identify the presence of the resistance genes *mef*A/E, *erm*B and *erm*A/TR, and also antibiotic susceptibility testing.

**Results.:**

The sample consisted of 348 GBS strains, 42 MLVA types were identified, and 4 of them represented 64 % of isolates. Serotype Ia was the most prevalent (42.2 %) and was found in a higher percentage associated with colonization, followed by serotypes V (24.4 %), II (17.8 %) and III (7.8 %). Serotype V was associated with invasive isolates and serotypes II and III with noninvasive isolates, without significant differences. All isolates were susceptible to penicillin. GBS 2018/ *hvg*A was observed in 17 isolates, with 11 belonging to serogroup III. The Hunter–Gaston diversity index was calculated as 0.879. The genes *mef*A/E, *erm*/B and *erm*/A/TR were found in 45, 19 and 46 isolates.

**Conclusion.:**

This report suggests that the circulating GBS belong to a limited number of genetic lineages. The most common genotypes were Ia/MT12 and V/MT18, which are associated with high resistance to macrolides and the presence of the genes *mef*A/E and *erm*A/TR. Penicillin remains the antibiotic of choice. Implementation of continuous surveillance of GBS infections will be essential to assess GBS epidemiology and develop accurate GBS prevention, especially strategies associated with vaccination.

## Introduction

Group B *Streptococcus* (GBS) is a commensal of the human digestive and genitourinary tracts, differentiated from other streptococci species by Rebecca Lancefield, who grouped them into serotypes according to the antigenic characteristics of capsular polysaccharides (CPS) [1]. From the 1940s it underwent evolutionary selection that made it better able to colonize healthy individuals and infect specific population groups [2]. In the 1960s it emerged as the most frequent cause of neonatal infection. The prevalence of maternal colonization in pregnancy varies between 10 and 40 %, and vertical transmission occurs in 40–70 % of newborns [3]. It is estimated that annually worldwide there are 319  000 cases of early and late neonatal sepsis, 90 000 neonatal deaths and 57 000 stilbirths due to streptococcal disease. Among survivors, up to 50 % evolve with neurodevelopmental impairment [4].

GBS is also associated with infection in non-pregnant adults. Underlying conditions such as diabetes mellitus, old age, obesity, cancer and compromised immunity make the disease more serious and prevalent. In this population, the clinical presentation can vary from soft tissue and skin infections through urinary tract infections to bacteraemia without a focus, reaching 10.9 cases per 100 000 population [5]. Less common clinical presentations such as meningitis, pneumonia and endocarditis worsen the prognosis. A new manifestation of GBS disease acquired from eating raw farmed freshwater fish was recently recognized, affecting non-pregnant and younger adults with fewer co-morbidities, suggesting greater virulence [6]. The burden of disease secondary to GBS infection is considered to be significant and of increasing prevalence [5, 7].

GBS strains are distinguished by differences in capsule polysaccharides into 10 different serotypes (Ia/Ib–IX). However, serotyping has low discriminatory power to investigate the epidemiological relationship of strains associated with maternal colonization and/or infections in pregnant women, neonates and non-pregnant adults [8, 9]. Among the tools for GBS epidemiological tracking, multiple-locus variable-number tandem repeat analysis (MLVA) is an easy, fast and low-cost methodology, with a higher discriminatory power compared to multilocus sequence typing (MLST) [10]. This method determines the number of tandem repeat sequences at different loci in a bacterial genome. It has been used for molecular characterization of other bacterial species [11] as well as for outbreak investigations [12]. In bacteria with a high degree of genetic diversity, such as GBS, the use of a small number of tandem repeats allows an accurate comparison among the allelic profile of the different isolates, allowing to determine whether epidemiologically related organisms are also genetically related [10, 13].

In the present study, the genetic diversity among GBS isolates from invasive or noninvasive disease and colonized pregnant woman was evaluated by MLVA. We also report serotype distribution, in addition to high rates of macrolide resistance associated with serotypes V and Ia, due to the presence of the *erm*A/TR and *mef*A/B genes, respectively. Knowledge of local epidemiological patterns may allow the improvement of measures for the prevention and treatment of streptococcal disease. Thus, the aim of this paper was to study the clustering of GBS isolates to identify possible genetic patterns associated with colonization or infection, according to the site and host from which the bacteria were recovered. The relationship between genogroups and serotypes, the prevalence of serotypes, the presence of the antimicrobial resistance genes *mef*A/E, *erm*B and *erm*A/TR, and resistance to the antimicrobials penicillin, vancomycin, tetracycline, clindamycin and erythromycin, were also evaluated.

## Methods

### Study population and bacterial isolates

The study was performed with a cross-sectional design, after approval by the research ethics committees of the institutions involved. The study included 348 consecutive, non-duplicated bacterial isolates obtained between August 2015 and January 2017 from inpatients and outpatients, non-pregnant adults, elderly patients, pregnant women and neonates/stilbirths, collected from sterile and non-sterile sites under the care of Laboratório de Biologia Molecular, Microbiologia e Sorologias (LBMMS/HU-UFSC). The strains were divided into invasive disease, defined as GBS isolated from a normally sterile site or from samples obtained from a non-sterile site in combination with clinical signs of infection; noninvasive urinary, defined as GBS isolated from urine; and colonizing, defined as GBS recovered from the rectovaginal tract.

Conventional methods as previously described [14] were used for culturing procedures and identification of the isolates as GBS. Isolates were stored at −80 °C in trypticase soy broth containing 20 % glycerol. All isolates were subcultured on 5 % sheep blood agar (Laborclin, Curitiba, Brazil) for 18 to 24 h at 35 °C in a 5 % CO_2_ atmosphere.

### Antimicrobial susceptibility testing

Antibiotic drug susceptibility testing was performed on Mueller–Hinton agar supplemented with 5 % sheep blood (bioMérieux, Rio de Janeiro, Brazil). The disc diffusion test was performed with clindamycin (2 µg), erythromycin (15 µg), penicillin (10U), vancomycin (30 µg) and tetracycline (30 µg) discs (Oxoid, Altrincham, UK). Isolates were investigated for inducible clindamycin resistance by double disc diffusion test to identify the cMLS (constitutive methylation), iMLS (inducible methylation) and M (efflux mechanism) resistance phenotypes. *Streptococcus pneumoniae* ATCC 49619 was used as a control. Tests were performed and interpreted according to Clinical and Laboratory Standards Institute (CLSI) standards (M100 S26) [15].

### Molecular capsular typing and the detection of the macrolide resistance gene and the *hvg*A surface protein

GBS genomic DNA was extracted by using the ReliaPrep Blood gDNA Miniprep System (Promega, Madison, WI, USA), in accordance with the manufacturer’s instructions. The capsular serotypes were determined by two multiplex PCRs, as previously described [16]. PCR was performed for gbs2018-ST17, a marker for the highly virulent GBS ST-17 clone, encoding the *hvg*A surface protein, following the protocol described by Lamy *et al*. [17]. Multiplex PCR was used to identify the *erm*B, *erm*A/TR and *mef*A/E genes from the GBS strains, using previously reported primers and conditions [18]. PCR was developed in 1.5 % agarose gel electrophoresis and stained with ethidium bromide. The products were visualized and documented using the Image Quant LAS 500 (GE Health Care, UK).

### MLVA

MLVA was performed as previously reported [9]. Six variable-number tandem repeats (VNTRs), termed SAGs (S= *Streptococcus *, AG=*agalactiae*), namely SAG2, SAG3, SAG4, SAG7, SAG21 and SAG22, were amplified by uniplex PCR reactions as previously described [10]. The number of repeats for each VNTR was determined by estimating the size of the amplicon separated in 2 % agarose gels (UltraPure Agarose, Invitrogen, Carlsbad, CA, USA), in which 100 bp (Ludwig Biotec, Alvorada, Brazil) and 50 bp (Bioline, Memphis, EUA) DNA ladders were included. The gels were photographed under ultraviolet illumination, using the Image Quant LAS 500 (GE Health Care, UK).

For *SAG*2, *SAG*3, *SAG*4 e *SAG*7, amplicons from 114 to 573 bp were identified. For *SAG*21 (48 bp per repetition unit) and *SAG22* (159 bp per repetition unit), amplicons with more than 1000 bp occured. The MLVA genotype of a strain was expressed as its allelic profile, corresponding to the number of repeats at each VNTR, listed in the order *SAG*2, *SAG*3, *SAG*4, *SAG*7, *SAG*21, *SAG*22. The number of repeats was determined by subtracting the offset (the number of nucleotides between the primers and the start/end of the repeat) and dividing the remaining number of nucleotides by the repeat length. After determining the allelic profile, the isolates were then grouped according to the lineage in the form of a phylogenetic tree by the eBURST program [19] to ascertain their heterogeneity. MLVA types (MTs) were numbered according to an in-house nomenclature. MTs sharing at least 60 % of similarity were included in the same MLVA cluster. The genetic diversity among the isolates was calculated using the Hunter–Gaston diversity index (DI) [20]. Analysis of MLVA numerical profiles was performed using BioNumerics v7.5 (Applied Maths, St-Martens-Latem, Belgium).

### Statistics

BioNumerics v7.5 software (Applied Maths, St-Martens-Latem, Belgium) was used for cluster analysis, using categorical values and the unweighted pair group method with arithmetic mean (UPGMA) algorithm, and for construction of dendrograms and minimum spanning trees (MSTs).

Statistical analyses were performed using the Statistical Package for the Social Sciences program (SPSS, version 22.0, IBM Brazil, São Paulo, SP, Brazil). The chi-square test was used to examine differences in serotype distribution between isolates recovered from infection and colonization specimens, as well as to evaluate correlation between erythromycin resistance phenotypes and genotypes. A *P*-value <0.05 was considered statistically significant.

## Results

Of the 348 GBS strains identified, 33 (9.5 %) invasive isolates were obtained from normally sterile site (blood, fluids, abscesses, soft tissue and stillbirth oropharynx), 60 (17.5 %) noninvasive isolates were obtained from the urinary tract and 255 (73.3 %) colonizing isolates were obtained from anogenital swabs and urine from healthy pregnant women.

Table 1 shows the number of isolates for each serotype, their clinical manifestations and patient group. Serotype Ia was the most prevalent (42.2%), followed by V (24.4%), II (17.8%) and III (7.8%). The serotypes showed a very similar distribution between different clinical manifestations. Serotype Ia was found in a higher percentage associated with colonization, serotype V was found in a higher percentage associated with invasive isolates, and serotypes II and III were found in a higher percentage associated with noninvasive isolates. These findings have no statistical significance.

**Table 1.** Serotype distribution of 348 GBS isolates among patient groups and clinical manifestations

Group	No. of isolates of serotype							
	Ia	Ib	II	III	IV	V	IX	Total
**Invasive isolates**								
Newborns/stillbirths*	5	–	2	1	–	–	1	9
**Adults**	4	2	5	1	–	6	–	18
**Pregnant**	2	–	–	–	–	–	–	2
**Elderly**	1	–	–	–	–	3	–	4
**Total (%**)	12 (36.4)	2 (6.1)	7 (21.2)	2 (6.1)	–	9 (27.7)	1 (3.0)	33
**Noninvasive urinary isolates**								
**Newborns**	–	–	2	–	–	–	–	1
**Adults**	10	–	7	4	–	3	–	24
**Pregnant**	3	–	2	2	–	3	–	10
**Elderly**	8	4	4	–	1	8	–	25
**Total**	21 (35.0)	4 (6.7)	14 (23.3)	6 (10)	1 (1.7)	14 (23.3)	–	60
**Colonizing isolates**								
**Pregnant**	114 (44.7)	8 (3.1)	41 (16.1)	19 (7.4)	7 (2.7)	62 (24.3)	4 (1.6)	255
**Total**								
**Total**	147 (42.2)	14 (4)	62 (17.8)	27 (7.8)	8 (2.3)	85 (24.4)	5 (1.4)	348

*Invasive isolates from a normally sterile site: two newborn haemocultures and seven stillbirth oropharynx swabs.

All GBS isolates were susceptible to penicillin and vancomycin. Resistance to tetracycline was found in 74 % of the GBS. Resistance to erythromycin was found in 26.4 % and clindamycin in 15.5 % of the samples, with 13.2 % expressing an inducible resistance phenotype. All clindamycin-resistant isolates were also resistant to erythromycin. iMLS was associated with the presence of the *erm*A/TR gene (84.8 %) and serotype V (71.7 %) (*P*=0.000). The cMLSB phenotype was found in eight isolates, seven of which carried the *erm*/B gene. Phenotype M was associated with the *mef*A/E gene (90.9 %) and serotype Ia (82.5 %) (*P*=0.001). Seven isolates showed both *erm*A/TR and *mef*A/B genes, six of them expressing the iMLS phenotype and one expressing the M phenotype. Two isolates amplified both *erm*B and *mef*A/B genes; one showed the M phenotype and the other was susceptible to erythromycin. The other susceptible isolates amplified macrolide resistance genes, four isolates amplified the *erm*B gene, five *erm*A/TR and six *mef*A/B. Two erythromycin-resistant isolates did not amplify any of the resistance genes investigated. These data are shown in Table 2.

**Table 2.** Erythromycin and clindamycin resistance, resistance phenotypes and macrolide resistance genes of 348 GBS isolates by serotype

Resistance	No. of isolates by serotype						
	Ia	Ib	II	III	IV	V	Total (%)
Erythromycin resistance	38	2	5	6	1	40	92 (26.4)
Clindamycin resistance	6	2	2	6	1	37	54 (15.5)
cMLS	2	–	–	2	–	4	8 (2.3)
iMLS	4	2	2	4	1	33	46 (13.2)
M	33	–	–	–	–	7	40 (11.5)
**Gene *mef*A/E**	36	–	1	–	–	8	45 (12.9)
**Gene *erm*B**	6	–	2	5	–	6	19 (5.5)
**Gene *erm*A/TR**	6	2	2	3	1	32	46 (13.2)

The VNTR loci investigated in the present study revealed the occurrence of 42 types among the 348 GBS strains, which were distributed among 25 clusters and 17 singletons. The main MTs are shown in Table 3. Four MTs represented 64 % of the isolates: MT 12 (25.4 %), MT 18 (14.4 %), MT 4 (12.4 %) and MT 26 (11.8 %). MTs 12 and 4 included 53.7 and 15.6 % of the serogroup Ia isolates, respectively; MTs 26 and 6 included 40.3 and 32.2 % of the serogroup II isolates, respectively; MT 18 included 48.2 % of the serogroup V and 37.5 % of the serogroup IV isolates; and MT 40 included 37 % of the serogroup III isolates. All five serotype IX isolates were included in MT 13. The clusters had a similar distribution among the different clinical manifestations and MT 26 was associated with noninvasive disease but not statistically significant. The combination GBS 2018/*hvg*A positivity (a marker of the hypervirulent clone ST-17) was observed in 17 isolates (4.9 %), 11 belonging to serogroup III (10 MT 40, 1 MT 41), 4 to serogroup Ia (2 MT 12, 1 MT 3, 1 MT 42), 1 to serogroup IV (MT 40) and 1 to serogroup V (MT 4). Among the *hvg*A-positive GBS strains, 15 were isolated from colonization and 2 from noninvasive disease, all in pregnant women. Table 3 shows the distribution characteristics of the MLVA types.

**Table 3.** Distribution of main MLVA types as a function of serotype, origin (invasive, noninvasive urinary, colonizing) and patient groups (adult, elderly, pregnant, neonate/stillbirth)

MLVA Group	Serotypes (Ia, Ib, II, III, IV, V); origin (ID, NI); patients group (Col, A, E, P, NS)													
	Total (%)	Ia	Ib	II	III	IV	V	ID	NI	Col	A	E	P	ns
**12**	87 (25.4)	79	–	1	2	1	4	7	16	64	11	4	69	3
**18**	50 (14.4)	4	–	1	1	3	**41**	5	5	40	4	6	40	–
**4**	42 (12.4)	23	2	3	1	–	13	4	6	32	3	2	35	2
**26**	41 (11.8)	2	–	25	7	1	6	3	17	21	10	7	22	2
**6**	23 (6.6)	2	–	20	–	–	1	2	1	20	3	–	20	–
**40**	12 (3.2)	–	–	–	10	1	1	–	1	11	–	–	12	–

Ia, Ib, II, III, IV, V, serotypes; ID, invasive disease; NI, noninvasive urinary disease; Col, colonizing; A, non-pregnant adults; E, elderly; P, pregnant women; NS, neonate/stillbirth.

**Fig. 1.**
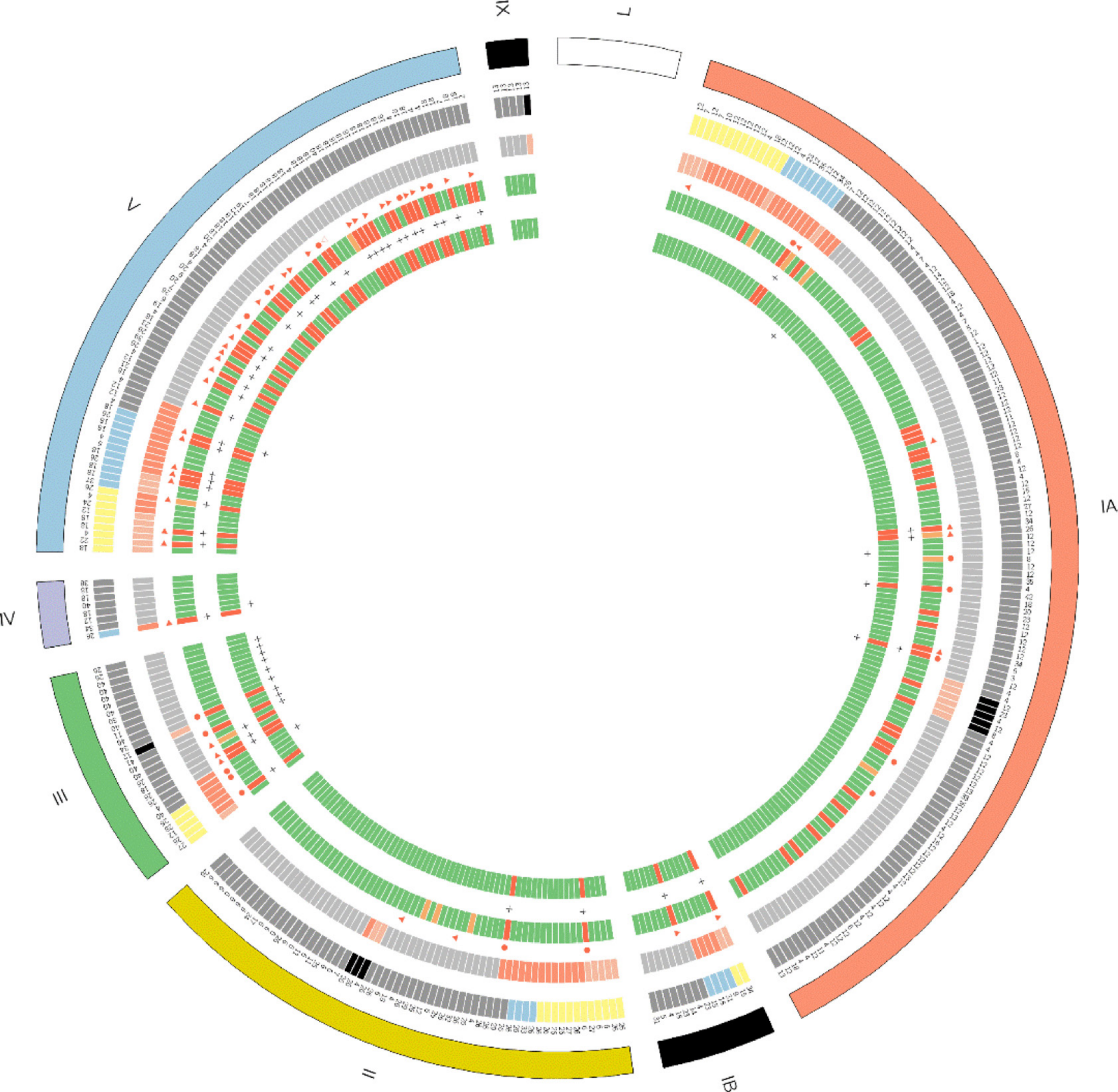
Graphical presentation of 348 GBS isolates constructed on the basis of MLVA patterns. From outside to inside: serotypes; MLVA types; patient groups (grey, pregnant women; black, neonates/stillbirths; yellow, adults; blue, elderly patients); clinical manifestations (grey, colonizing isolates; red, noninvasive urinary isolates; orange, invasive isolates); orange triangle, PCR-positive *erm*/TR gene; orange circle, PCR-positive *erm*/B gene; susceptibility to erithromycyn (green, susceptibility; red, resistance); d-test-positive; susceptibility to clindamycin (green, susceptibility; red, resistance), GBS 2018 *hvg*A-positive.

Assessing resistance to erythromycin, 65 % of resistant isolates were included in three MTs: 27 % in MT 18, 23 % in MT 12 and 14.1 % in MT 26. Two MTs included 65.5 % of isolates resistant to clindamycin: 44.4 % were in MT 18 and 24.1 % were in MT 26. The *mef*A/B gene was predominant in MT 12 (48.9 %) and the *erm*/TR gene in MTs 18 (43.5 %) and 26 (30.4 %), while the *erm*/B gene was distributed among several MTs. The Gaston–Hunter DI was calculated at 0.879. Fig. 1 shows the graphical presentation of 348 GBS isolates constructed based on MLVA patterns.

## Discussion

This report presents a molecular evaluation of a large number of GBS strains recovered from different sites and clinical settings in southern Brazil. Among the colonization samples, serotype Ia represented 44.7 % of isolates, around the 50 % described by Russell *et al*. [21]. In our sample, serotype V stood out as the second most prevalent. Serotype V was the most prevalent in invasive adults isolates in the USA, reaching 30 % of isolates in 2009, but has shown a decreasing prevalence in recent years, comprising only 18 % of isolates in 2016 [22, 23]. The high prevalence of serotype V in this study is justified because they mainly originate from colonizing isolates. Our study has a lower prevalence of serotype III than has been described for South America [21], probably due to lack of inclusion of late onset diseases (LOD) cases [24–26].

The adhesin *hvg*A specific for the hyper-virulent clone ST17 was found in 10 serotype III isolates and 1 serotype IV isolate. The emergence of serotype IV has been described and seems to be due to a switch from CC17 hyper-virulent serotype III to serotype IV [27]. The reason for the low prevalence of serotype IV among the *hvg*A isolates found in this research is unclear, but may correspond to local bacterial diversification, local differences in human host immune response or over-representation of colonizing isolates.

According to the distribution of serotypes, in the samples studied the effectiveness of a vaccine against capsular polysaccharides (CPS) could change from 54 to 98.5 %, depending on the number of serotypes covered [28]. The emergence of GBS serotype IV disease in adults [22, 29], capsular switching, mobile DNA element changes and the plasticity of the genome [30] are matters of concern, hence serotypes and virulence markers should be checked constantly. The absence of isolates with the non-typeable phenotype suggests the superiority of multiplex PCR over latex agglutination [8, 9, 31].

In our samples, the 60 % cut-off point for similarity was selected to allow for more comparisons with other populations, and 42 distinct MTs were identified, with a DI of 0.879. Using a collection of 186 strains isolated from humans and cattle, with a cut-off point of 50%, Haguenoer *et al*. described nine MTs and a DI 0.960 [10]. Otaguiri *et al*. evaluated 83 isolates from female colonization cases, and applying a cut-off point of 85 % reported 15 MTs with a DI 0.840; and Radtke *et al.*, studying 126 isolates, mostly of human invasive and international reference strains, found 70 MT and a DI 0.840, with cut-off point not described [13, 32]. Methodological differences make it difficult to compare such studies, but the large number of isolates should be considered a strength.

In the current study, 64 % of the isolates belonged to four clonal groups, suggesting that circulating GBS belong to a limited number of genetic lineages. In the Haguenoer *et al*. sample, the four largest MT encompassed 80 % of the isolates, the largest of which included 59 (31 %) isolates [9], and in the Otaguiri *et al*. sample the four largest MTs contained 71 % of the isolates, with the largest comprising 26 (33.7 %) isolates [32].

Penicillin-non-susceptible GBS are extremely rare [33, 34]. In this study, all strains remained susceptible, confirming its effectiveness for first-line use [33]. While resistance can reach 43.9 % for clindamycin and 55 % for erythromycin [23, 34], in this study resistance was found in 15.5 and 26.4 % of isolates, respectively. However, our resistance rates were higher than the rates referred by other Brazilian researchers, who have reported variations from 1.9–16.7 % for clindamycin and 4–13.2 % for erythromycin [35–37]. In the USA, erythromycin resistance was 1.8 % in the 1980s [29], and this drug is no longer recommended today [34]. Indiscriminate use of antimicrobials, an increasingly old population with comorbidities, and agricultural antibiotic usage are possibly associated with increased resistance [25, 29, 30]. First-line antimicrobials were not affected by this phenomenon, but resistance to macrolides is a problem for allergic patients [35, 38–40].

One of objectives of this investigation was to determine the genetic basis of erythromycin resistance. Among the 92 macrolide-resistant isolates described in the present study, 43.5 % [40] belonged to serotype V. It has emerged as a cause of human disease, and was the serotype most associated with erythromycin resistance in several studies [41, 42], mostly carriyng the *erm*A/TR gene [32, 43] or the *erm*B gene [43–45]. Da Cunha *et al*. reported that macrolide resistance contributed to CC1 expansion, and was predominantly composed of serotype V isolates [2]. The M phenotype was the second most prevalent isolate in this study, and was associated with the *mef*A/E gene (90.9 %) and serotype Ia (82.5 %), with 58.3 % [21] belonging to MT 12. An association between efflux pump-mediated resistance to erythromycin encoded by the *mef*A/E gene and serotype Ia has been described in other studies [32, 42, 44]. However, a previous study conducted with GBS serotype Ia from different Brazilian regions did not detect resistance to macrolides [46]. In our study, the main resistance mechanisms observed, the *erm*A/TR and *mef*A/E genes, were significantly associated with MTs 18 and 12, respectively, and such isolates also showed resistance to tetracycline. Furthermore, the emergence of a new clone family that carries resistance to antimicrobials widely used in prophylaxis and therapy, like erythromycin and clindamycin, may have major consequences for clinical practice [39].

In conclusion, this report of genotypic diversity by MLVA study of GBS isolates in southern Brazil suggests that circulating GBS belong to a limited number of genetic lineages. The most common genotypes circulating were Ia/MT12 and V/MT18, and seem to be associated with high resistance to macrolides, especially due to the presence of the genes *mef*A/E and *erm*A/TR, when compared to other Brazilian regions. Continuous surveillance of infections will be essential to assess GBS epidemiology and develop accurate disease prevention, especially strategies associated with vaccination.
